# ssDNA Wrapping of Single-Walled Carbon Nanotubes and the Role of Sequence Periodicity: A Critical Review

**DOI:** 10.3390/ijms27219655

**Published:** 2026-10-28

**Authors:** Lubanah Al Nissafi, Moussa Emjeida, Raja Venkatesan, Alexandre A. Vetcher

**Affiliations:** 1 Institute of Pharmacy and Biotechnology, RUDN University, 6 Miklukho-Maklaya St., Moscow 117198, Russia; moussaemjeida88@gmail.com (M.E.); avetcher@gmail.com (A.A.V.); 2 School of Chemical Engineering, Yeungnam University, 280 Daehak-Ro, Gyeongsan 38541, Republic of Korea

**Keywords:** single-walled carbon nanotubes (SWCNTs), chirality (n, m), single-stranded DNA, wrapping mechanism, wrapping pitch, recognition sequences, natural periodic sequences, tandem repeats, genome structure, nanosensing

## Abstract

Noncovalent hybrids of single-stranded DNA (ssDNA) and single-walled carbon nanotubes (SWCNTs) are central to chiral sorting and near-infrared biosensing, yet no quantitative rule connects the periodicity of a natural sequence to the geometry of a given nanotube. Earlier reviews treat the sequence as a tool for sorting or sensing; this critical review asks what the published evidence up to 2026 establishes about periodicity. We compare base–wall affinities from calculation, calorimetry and force spectroscopy; the periods reported by cryo-electron microscopy, scanning tunnelling microscopy, atomic force microscopy (AFM) and X-ray scattering in solution; the recognition sequences used for single-chirality sorting; and the few studies of genomic DNA. The experimental order of base affinities depends on the method and on whether the surface is flat or curved. Helical pitches of 1.6–3.3 nm, spacings of 8.6–14.3 nm between strands in solution and the AFM period of about 14 nm are different quantities. Recognition depends on sequence pattern, length, chiral angle and handedness in addition to diameter. To our knowledge, base spacing at fixed composition has been varied only in covalently cross-linked hybrids; for noncovalent wrapping, this test is still missing. We outline the next experiments and the reporting standards and open data they require.

## 1. Introduction

Single-walled carbon nanotubes (SWCNTs) combine high electrical conductivity and mechanical strength [1,2] with a stable near-infrared (NIR) emission whose transitions can be assigned to individual (n, m) species [3]. Because this emission falls within the transparency window of biological tissue, the tubes have become an attractive platform for noninvasive sensing and imaging [4,5,6,7,8] in addition to their use in transistors, flexible sensors and transparent conductors [2,9,10]. By 2013, the worldwide capacity for producing carbon nanotubes already exceeded several thousand tonnes per year, and nanotube powders were being added to commercial products such as rechargeable batteries, automotive parts, sports equipment, boat hulls and water filters [11]. For SWCNTs, advances in scalable synthesis remain critical for many commercial applications [12]. Their combination with DNA dates back to 2003, when single-stranded DNA (ssDNA) was shown to disperse SWCNTs under ultrasonication, and modelling pointed to helical wrapping through π–π stacking [13]. In the same year, the binding was shown to depend on sequence, so that tubes wrapped by (GT)_n_ could be separated by diameter and electronic type [14]. A third early result came from spectroscopy. Using surface-enhanced infrared absorption (SEIRA), Dovbeshko et al. [15] recorded changes in the DNA backbone on binding to SWCNTs, which they interpreted as an A-to-B transition; graphite powder had only a slight effect. This result indicated that binding to the nanotube also changes the conformation of the DNA. Since then, the libraries of recognition sequences have grown [16,17,18,19], and the structure of the wrap has recently been determined at sub-nanometre resolution [20]. The molecular basis of recognition, however, is still not fully understood [21].

**Figure 1:**
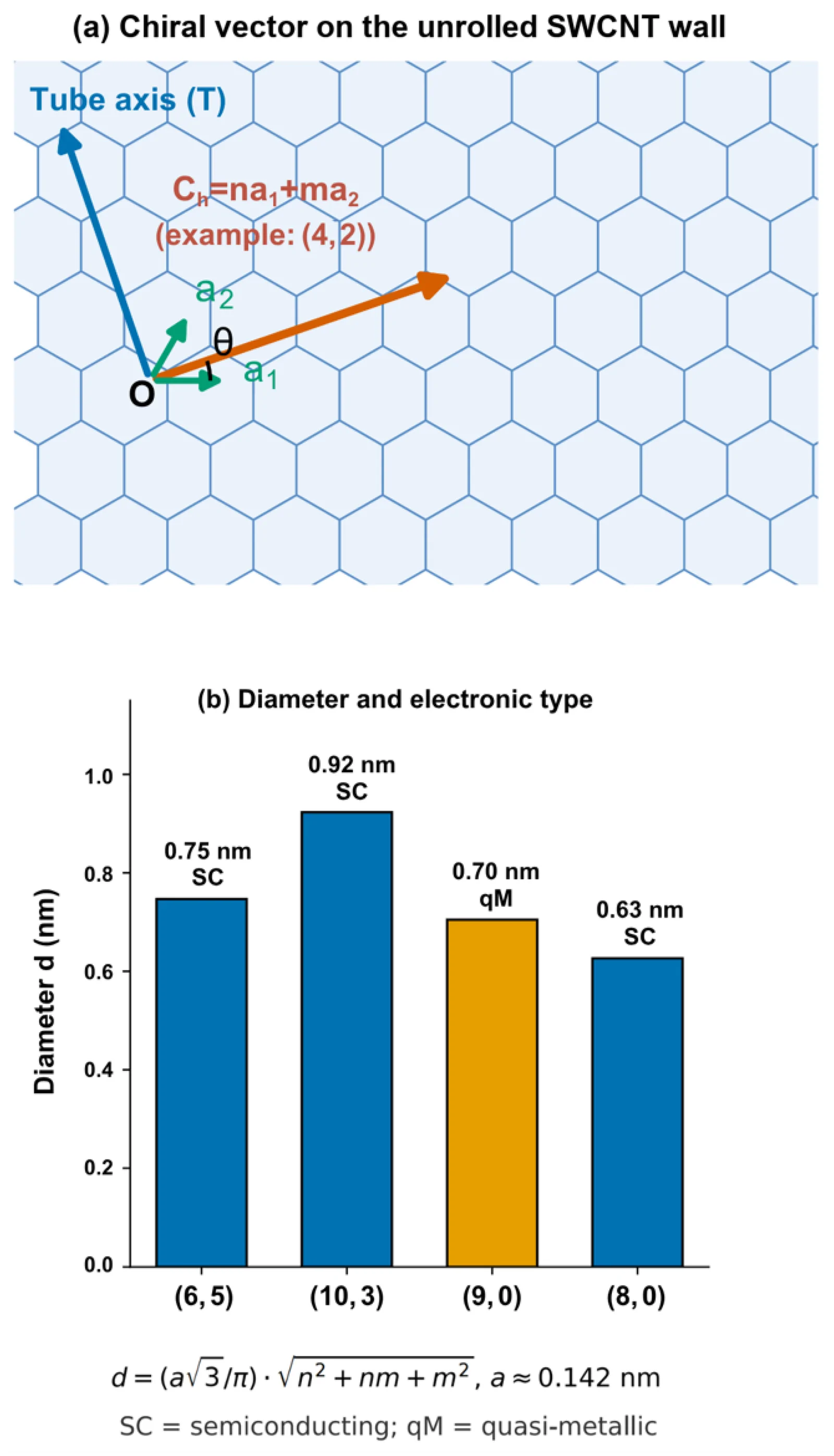
Geometry of a single-walled carbon nanotube (SWCNT) and its link to electronic type. (**a**) The unrolled SWCNT wall: the chiral vector C_h_ = n·a_1_ + m·a_2_ sets the direction along which the carbon lattice rolls into a cylinder; a_1_ and a_2_ are the lattice basis vectors, θ the chiral angle (measured from the zigzag direction a_1_), and T the tube axis, perpendicular to C_h_. (**b**) Diameter *d* = (*a*√3/π)·√(*n*^2^ + *nm* + *m*^2^) (*a* ≈ 0.142 nm) and the electronic type set by (*n* − *m*) mod 3 for four representative chiralities: (6, 5) ≈ 0.75 nm and (10, 3) ≈ 0.92 nm are semiconducting (SC); (9, 0) ≈ 0.70 nm is quasi-metallic (qM); (8, 0) ≈ 0.63 nm is small-diameter semiconducting. Illustration prepared by the authors (matplotlib); it shows standard nanotube geometry and contains no new data.

The electronic character of a nanotube and the shape of its DNA hybrid both follow from its chirality, expressed as the index pair (n, m). The two integers fix the diameter and the chiral angle, and with them, the curvature of the surface to which the DNA strand must conform (Figure 1). Almost all work so far has used synthetic oligonucleotides of fixed design, with the (GT)_n_ repeats as the standard example [14]. Yet the (GT)_n_ motif is itself a dinucleotide microsatellite, and genomic DNA has been shown to wrap nanotubes helically [22] and to enrich the (6, 5) species [23]. Many of the main results of the field were, thus, obtained with a natural periodic motif, although, as far as we know, periodicity has not been studied as a principle in its own right.

This review examines that gap. Its main question is whether a SWCNT of given geometry can distinguish the fine structural differences among natural periodic sequences. At first glance, these repeats look alike, since all of them are periodic and built from a few motifs, but they differ in composition, in period and in their tendency to fold into secondary structures. More specifically, we ask whether these differences alter the packing of the strands on the carbon surface strongly enough for a nanotube to tell them apart by its optical and conformational response. We restrict the scope to SWCNTs, because graphene and other carbon substrates raise different questions and need separate treatment. Our aim is to give a critical account of the wrapping mechanisms and to identify which parts of the question the published data already answer and which still need a direct test.

Because periodicity is the central concept of this review, we define it at the outset. A sequence is periodic with period *p* when a motif of *p* nucleotides is repeated in tandem, so that, apart from the ends, the base at position *i* is the same as the base at position *i* + *p*. The period is 2 for (GT)_n_, 3 for (TAT)_n_ and 6 for the human telomeric repeat (TTAGGG)_n_, and the number of copies, *n*, sets the length. Natural tandem repeats are classed by the length of the repeat unit into microsatellites, minisatellites and satellites (Section 3.2), and their copies are not always identical. Periodicity, in this sense, is a property of the primary sequence. It is distinct from composition, the proportion of each base, which a periodic motif shares with all of its permutations. It is also distinct from the three lengths of the wrap compared in Section 4.3: the helical pitch of one strand, the spacing between neighbouring strands and the apparent period seen in earlier atomic force microscopy. These lengths are measured in nanometres along the tube, whereas the period of a sequence is counted in nucleotides along the strand. We also do not use the term for a property that varies periodically with the length of a sequence (Section 4.4). Whether periodicity matters thus comes down to whether the period *p*, at fixed composition and length, changes the way a strand packs on a given nanotube.

Several reviews already cover parts of this field. Zheng reviewed the sorting of nanotubes by DNA and its basis in differences of solvation [24], and Yang et al. reviewed it together with the growth and characterisation of chirality-pure tubes [25]. A recent perspective summarises the progress and mechanisms of DNA-mediated sorting [26]. The optical biosensing applications of DNA-wrapped and other functionalised nanotubes have been reviewed several times [4,5,27], as has the design of sensors with biological recognition elements [28]. In all of these, the DNA sequence is treated mainly as a tool for sorting or sensing. To our knowledge, none of them compares the base-affinity data obtained by different methods or separates the pitch of the wrap from the longer periods measured by other techniques. Nor do they ask whether the periodicity of natural repeats is itself a variable that a nanotube can read. The present review addresses these three points and, because a test of periodicity depends on comparable data, also considers reproducibility between laboratories, reporting standards and open data.

## 2. Scope and Approach of the Review

This is a narrative critical review rather than a systematic one, and it does not claim exhaustive coverage. We assembled the literature by starting from the foundational papers of the field [13,14,16] and from recent reviews [4,5,24,25] and by following their citations backwards and forwards. The review covers work published up to September 2026. For the period 2023–2026, the search was extended in the OpenAlex database by title queries that combined nanotube with DNA, oligonucleotide, genomic, telomeric, quadruplex, repeat, sorting, separation, chirality, biosensing or fluorescent, and by a keyword search for review articles on DNA–nanotube hybrids. These queries returned 905 unique records, which were screened by title and abstract. The screening, together with general web searches and citation tracking, added 21 studies published in 2023–2026, and their bibliographic data were taken from Crossref. Priority went to primary studies that report structural, thermodynamic or optical data on noncovalent ssDNA–SWCNT hybrids. Reviews and work on other dispersants or on nanotube electronics are cited only for context. In each primary study, we noted the type of sequence (natural periodic, synthetic periodic, homopolymer or random), the motif and its period, the nanotube species, the quantity measured and the method. Numerical values in the tables are quoted as reported in the cited papers, and values obtained by different methods are kept apart throughout. The bibliographic details of every reference were checked against the publishers’ records. A generative AI tool assisted with parts of this work (see Acknowledgments); all outputs were checked by the authors against the original sources. Table 1 outlines the main stages through which the field has passed.

## 3. The Two Partners: Nanotube Geometry and Periodic DNA

### 3.1. Nanotube Geometry and Handedness

The indices (n, m) define the geometry of a nanotube, and the behaviour of the hybrid depends on that geometry. They give the diameter, *d* = (*a*√3/π)·√(*n*^2^ + *nm* + *m*^2^), with *a* ≈ 0.142 nm the C–C bond length, and the chiral angle, *θ* = arctan[√3·*m*/(2*n* + *m*)] (Figure 1a). The electronic type follows from the same indices. A tube is metallic or quasi-metallic if (*n* − *m*) is a multiple of three and semiconducting otherwise (Figure 1b). Every chiral tube also exists as a left-handed and a right-handed enantiomer. The indices do not show this degree of freedom, but it matters for a chiral polymer such as DNA [17]. Only the semiconducting species fluoresce, each at its own absorption and emission wavelengths, so the composition of a sample can be read optically [3].

The species that recur in the studies reviewed here span only a narrow range of diameters, from 0.75 nm for (6, 5) and (9, 1) to about 1.0 nm for (8, 7) and (10, 5). The range reflects the small-diameter materials that are usually dispersed, so it should not be read as a geometric optimum of the hybrid. Nor is DNA the only route to structure-pure material. Conjugated polymers extract semiconducting tubes selectively in organic solvents [52,53,54,55,56], and solid catalysts can bias chirality during growth [57]. The advantage of DNA is that it works in water and that its selectivity is encoded in a sequence, which can be searched and varied [16,24].

### 3.2. Natural Periodic Sequences: What They Share and Where They Part

Tandem repeats (satellites, minisatellites and microsatellites) and dispersed repeats such as transposable elements together make up more than half of the human genome [58,59], and by one estimate, about two-thirds [60]. They have structural and regulatory roles in chromatin organisation and in the control of gene expression [59,61]. What tandem repeats share is periodicity, since one motif is repeated faithfully over a long stretch. They differ in the composition of the motif (G/A-rich versus T/C-rich), in its length and period, in their tendency to fold into secondary structures and in how much the repeat number varies from one allele to another [58,61]. The best-known repeat that folds is the human telomeric repeat (TTAGGG)_n_ [62], whose G-rich strand forms G-quadruplexes [63] and whose C-rich strand forms an i-motif [64].

These differences make natural repeats a suitable test system for discrimination, provided that two kinds of difference are kept apart (Section 1). Compositional effects depend on the proportion of the bases, for example, on the G/C versus A/T content. Periodicity effects depend on the length of the repeat unit and on the spacing of identical bases along the strand at fixed composition. If the packing of ssDNA on a SWCNT depends on the period and composition of the motif, sequences from a similar alphabet but of different periodicities should give distinguishable responses. The relevant form is the single strand. Its bases are free to stack on the nanotube wall, whereas in a duplex, they are paired and stacked inside the helix, and long genomic DNA disperses nanotubes efficiently only after the complementary strand has been removed [22].

There are several reasons to focus on natural periodic sequences rather than on arbitrary synthetic oligomers. The main one is that periodicity is the feature on which any geometric mechanism of recognition would have to rely. A regular motif repeats the same contacts at fixed intervals along the strand, so its fit to the circumference of a given tube could build up over the whole length. In an aperiodic sequence, by contrast, the contacts change from one turn to the next. A regular wrap, as such, does not require a periodic sequence, since long genomic strands of effectively random sequence also wrap nanotubes in regular helices [22] (Section 5.4). What remains open is whether a repeated pattern of bases adds selectivity to that wrap. Binding strength, for its part, already depends on the exact length of a repeat, down to a single nucleotide [38,39] (Section 4.4). Whether such differences add up along a long periodic run, and so, separate motifs of similar compositions, has not been tested. On the biological side, repeats make up a large part of the genome [58,59], so a method that distinguishes repeats on a nanotube could, in principle, be applied to genomic repeats as well. Finally, there is a precedent, because the canonical sorting sequence (GT)_n_ is itself a natural dinucleotide microsatellite.

The argument is also practical. Long repeats can be amplified from natural or cloned templates, whereas direct solid-phase synthesis loses fidelity as length grows [65]. Gigliotti et al. made the same point for nanotubes, noting that sequences designed for wrapping have to be synthesised and work best below about 150 bases, while genomic single strands far longer than that disperse nanotubes efficiently [22].

## 4. How ssDNA Binds and Wraps a Nanotube

### 4.1. The Wrapping Mechanism

**Figure 2:**
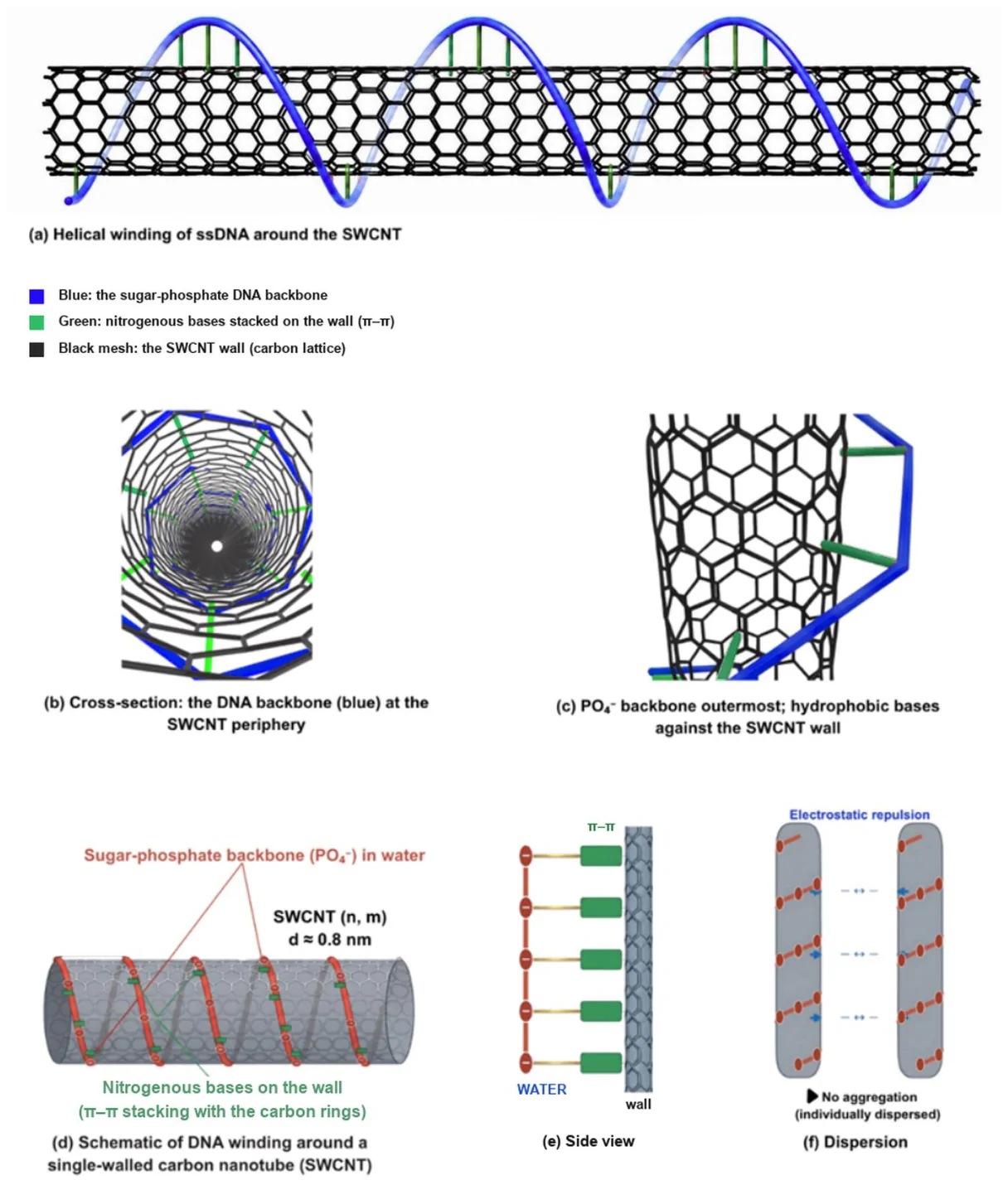
Molecular mechanism of ssDNA wrapping on the SWCNT surface. (**a**–**c**) Three-dimensional illustration drawn in Blender: (**a**) a single strand wound helically around the tube; (**b**) view along the tube axis, with the backbone (blue) at the periphery; (**c**) close-up of the bases (green) stacked on the carbon wall, with the backbone facing outward. (**d**–**f**) Schematic drawings: (**d**) the strand on a tube of about 0.8 nm diameter, with the bases (green) on the wall and the sugar–phosphate backbone, which carries one negative charge per nucleotide (red, “−” marks), in the water; (**e**) side view of the same arrangement; (**f**) electrostatic repulsion between the charged coatings of neighbouring tubes (blue arrows) keeps the tubes individually dispersed. In this way, ssDNA disperses SWCNTs without altering their covalent structure. Schematic illustration (not to scale) prepared by the authors; panels (**a**–**c**) are not the result of a simulation or calculation, and the figure contains no new data.

The hybrid forms because the free energy of the system decreases. In water, bare SWCNTs aggregate because their surface is hydrophobic, and a DNA strand, with hydrophobic bases on a hydrophilic phosphate backbone, acts as the amphiphilic intermediary. The process can be described in three stages (Figure 2). The bases first adsorb onto the carbon wall by π–π stacking between the aromatic rings [13,66]. The strand then wraps around the tube. In all-atom simulations, it winds spontaneously into compact helices within a few nanoseconds, and the simulations trace this behaviour to electrostatic and torsional forces in the sugar–phosphate backbone [66]. Finally, the hybrid is stabilised in solution, as described below.

The sugar–phosphate backbone links the nucleotides into a continuous chain. Once the strand is wrapped, the backbone turns outward toward the water while the hydrophobic bases lie flat against the wall. The backbone carries regularly spaced PO_4_^−^ groups, one negative charge per nucleotide, and these charges raise an electrostatic barrier between neighbouring tubes that prevents aggregation. The measured charge density of poly(GT) hybrids is 6.0 and 6.8 electrons per nanometre on (6, 5) and (7, 5) tubes, and their electrophoretic mobility is comparable to that of double-stranded DNA [67]. In this respect, ssDNA behaves like a surfactant, a dispersing agent that leaves the covalent structure of the tube unchanged and keeps it individually dispersed (Figure 2).

However, π–π stacking is only one of several forces that decide how stable the hybrid is. Stacking of the bases on the wall supplies most of the binding energy. The binding free energy of ssDNA to a nanotube was estimated early on to rival that of two nanotubes for each other [13], and simulations find the bases lying flat on the surface and strongly aligned [68]. Not all bases are in contact with the wall, though; a significant fraction unstacks at room temperature, which lowers the effective adhesion per base [69]. Electrostatic repulsion between the backbone charges opposes close packing, and at low ionic strength, the balance between adhesion and repulsion is enough to select an optimal helical geometry [69]. Hydrogen bonds between bases, within one strand, and between neighbouring strands add a third contribution and stabilise the ordered wraps of recognition sequences [20,36,37]. Stacking and the hydrophobic effect thus provide the binding energy, electrostatics set the spacing, and hydrogen bonding orders the wraps of recognition sequences (Table 2).

### 4.2. Base–Wall Affinity in Calculation and Experiment

Any discussion of sequence dependence starts from the binding strength of the individual bases, and on this point, the published data are not consistent (Table 3). Density-functional theory (DFT) calculations for graphene, taken as the flat limit of the nanotube wall, agree that guanine binds most strongly, but the order of the other three bases depends on how dispersion is treated. An early DFT study found G > A ≈ T ≈ C [31]. With the van der Waals-corrected Perdew–Burke–Ernzerhof functional (PBE + vdW), the Tkatchenko–Scheffler (TS) scheme, the van der Waals density functional (vdW-DF) and Grimme’s empirical dispersion correction (DFT-D2), the order is G > A > T > C [70,71]. The later version of this correction (DFT-D3) and the second version of the van der Waals density functional (vdW-DF2) give G > A > T ≈ C [71]. The absolute binding energies also depend on the functional, from 0.54–0.75 eV in the local-density approximation (LDA) to 0.93–1.18 eV with PBE + vdW for the four bases on graphene and boron nitride sheets [70]. For a (5, 5) nanotube, Hartree–Fock calculations combined with a classical force field give G > A > T > C without solvent and G > T > A > C once the solvation energy is included [32].

**Table 3:** Order of base binding to graphitic surfaces reported by different methods.

Surface and Medium	Method	Order Reported	Source
Graphene, vacuum	DFT with additional second-order Møller–Plesset (MP2) calculations	G > A ≈ T ≈ C	[31]
Graphene, vacuum	DFT with dispersion (PBE + vdW, TS, vdW-DF, DFT-D2)	G > A > T > C	[70,71]
Graphene, vacuum	DFT with dispersion (DFT-D3, vdW-DF2)	G > A > T ≈ C	[71]
(5, 5) SWCNT, vacuum	Hartree–Fock with a classical force field	G > A > T > C	[32]
(5, 5) SWCNT, with solvation energy	Hartree–Fock with a classical force field	G > T > A > C	[32]
Graphite, water	Adsorption isotherms of the free bases	G > A > T > C	[72]
Graphene, water	Isothermal titration calorimetry	G > A > C > T (C and T close)	[73]
SWCNT, water	Isothermal titration calorimetry	T > A > C	[32]
Graphite, water	Force spectroscopy of homopolymers	T > A > G ≥ C	[74]
SWCNT, water	Force spectroscopy of homopolymers	A > G > T > C	[74]

**Figure 3:**
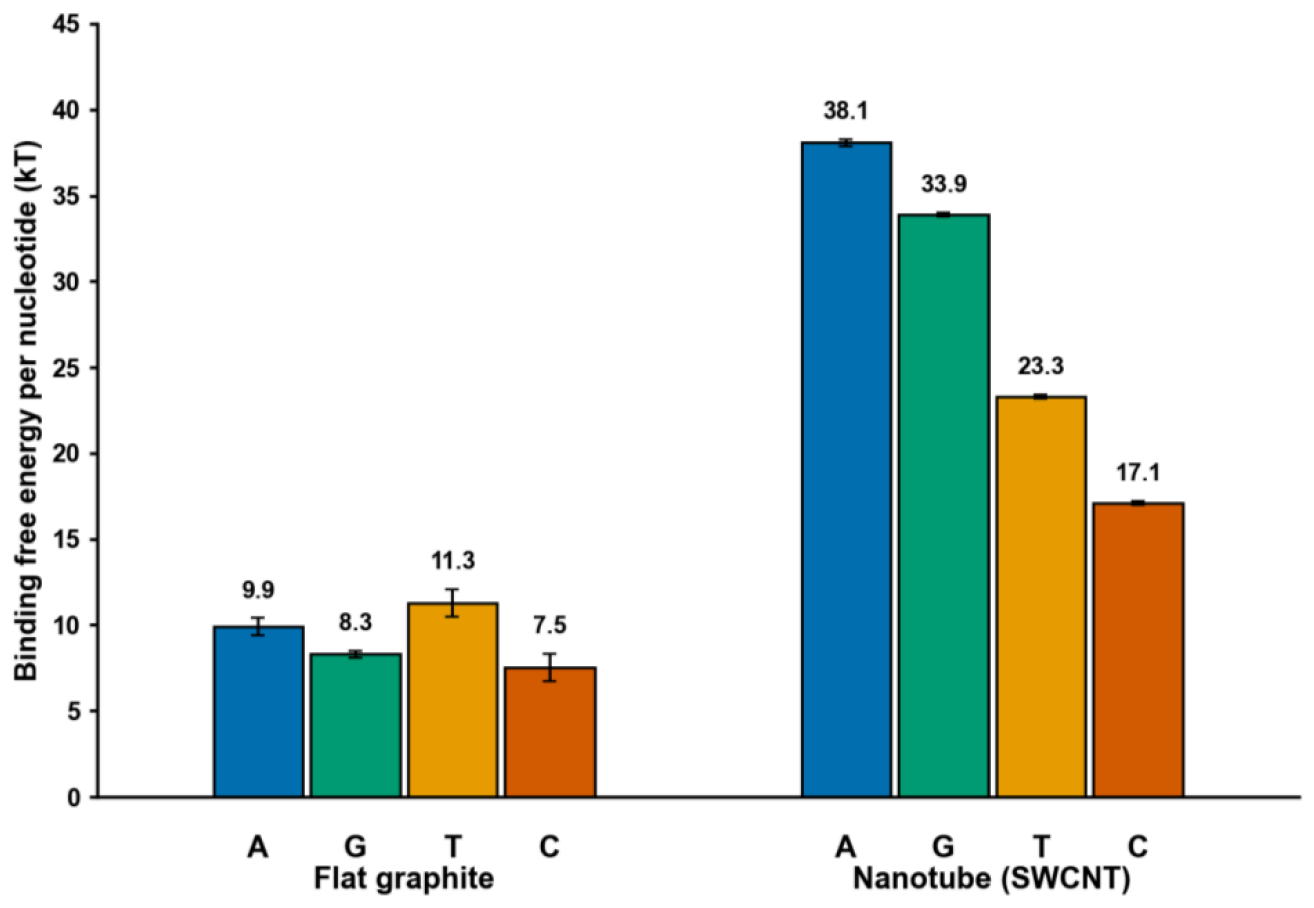
Binding free energy per nucleotide of ssDNA homopolymers on flat graphite and on SWCNTs measured by single-molecule force spectroscopy and expressed in units of the thermal energy kT. The curved nanotube wall binds every homopolymer more strongly than graphite and ranks the bases in a different order. All values and error bars (the uncertainties given in the source) are taken from the published study [74] and replotted by the authors (matplotlib); no new data are shown. Pairwise statistical comparisons of these values are given in Section 4.2.

Experiments in water do not give a single order either. Adsorption isotherms on crystalline graphite rank the bases G > A > T > C [72]. Calorimetry on graphene gives G > A > C > T, with C and T close enough to be interchangeable [73], and calorimetry on nanotubes gives T > A > C [32]. Single-molecule force spectroscopy differs from these methods in that it measures the binding free energy per nucleotide of homopolymers rather than of free bases in units of the thermal energy kT. On graphite, it ranks poly(T) > poly(A) > poly(G) ≥ poly(C). An early peeling study found 11.5 and 8.3 kT for thymine and cytosine nucleotides and a later one 11.3, 9.9, 8.3 and 7.5 kT for T, A, G and C [33,74]. On nanotubes, the same technique gives a different order and far larger values: poly(A) > poly(G) > poly(T) > poly(C) at 38.1, 33.9, 23.3 and 17.1 kT per nucleotide [74] (Figure 3).

A full analysis of variance would require the individual measurements, but the reported means and uncertainties allow pairwise comparisons. We treated the reported uncertainties as standard errors and applied a Holm–Bonferroni correction to the 16 comparisons in Figure 3; all *p* values given below are adjusted. Every homopolymer binds significantly more strongly to the nanotube than to graphite, and all four homopolymers differ from one another on the nanotube (*p* < 10^−6^ in each case). On graphite, only two groups are resolved. Poly(T) and poly(A) bind more strongly than poly(G) and poly(C) (*p* ≤ 0.033), whereas poly(T) and poly(A) (*p* = 0.28), and poly(G) and poly(C) (*p* = 0.33), cannot be distinguished at the level of the reported uncertainties. The reversal between the two surfaces is, nevertheless, significant, because thymine binds more strongly than guanine on graphite but more weakly on the nanotube.

Two conclusions follow for this review. The flat surface is not a good model of the tube, since binding to the curved wall is several times stronger than to graphite and ranks the bases differently [74]. Affinities calculated or measured on graphene therefore cannot be transferred directly to nanotubes. Free bases and bases held in a strand also do not behave alike, because the backbone limits which bases can stack at the same time [33,69]. The frequently cited solvated order G > T > A > C [32] is, thus, only one of several results obtained for one armchair tube with one method, and it should not be considered established. The data support a more limited statement. Adenine and guanine bind more strongly than cytosine in each nanotube experiment that included them, and thymine, the partner of guanine in the (GT)_n_ repeats, binds more strongly than cytosine in both [32,74]. At the level of whole sequences, a selection from a random library found that particular base compositions and base patterns govern affinity, with hydrogen bonding between bases contributing to the stability of the adsorbed strand [49].

In short, three physical differences between the methods explain most of these discrepancies. Calculations in vacuum omit the solvent, whereas in water, the solvation of the bases and the hydrophobic effect contribute to binding and can reorder the bases, as one calculation with and without the solvation energy shows [32]. The curvature of the wall changes the interaction itself: force spectroscopy combined with simulations found that ssDNA binds the curved nanotube far more strongly than flat graphite and attributed the difference to the spontaneous curvature of ssDNA [74]. Finally, bases held in a strand are constrained by the backbone and by their neighbours, so the affinities of free bases do not transfer directly to polymers [33,69].

### 4.3. The Structure of the Wrap: Pitch and Handedness

**Figure 4:**
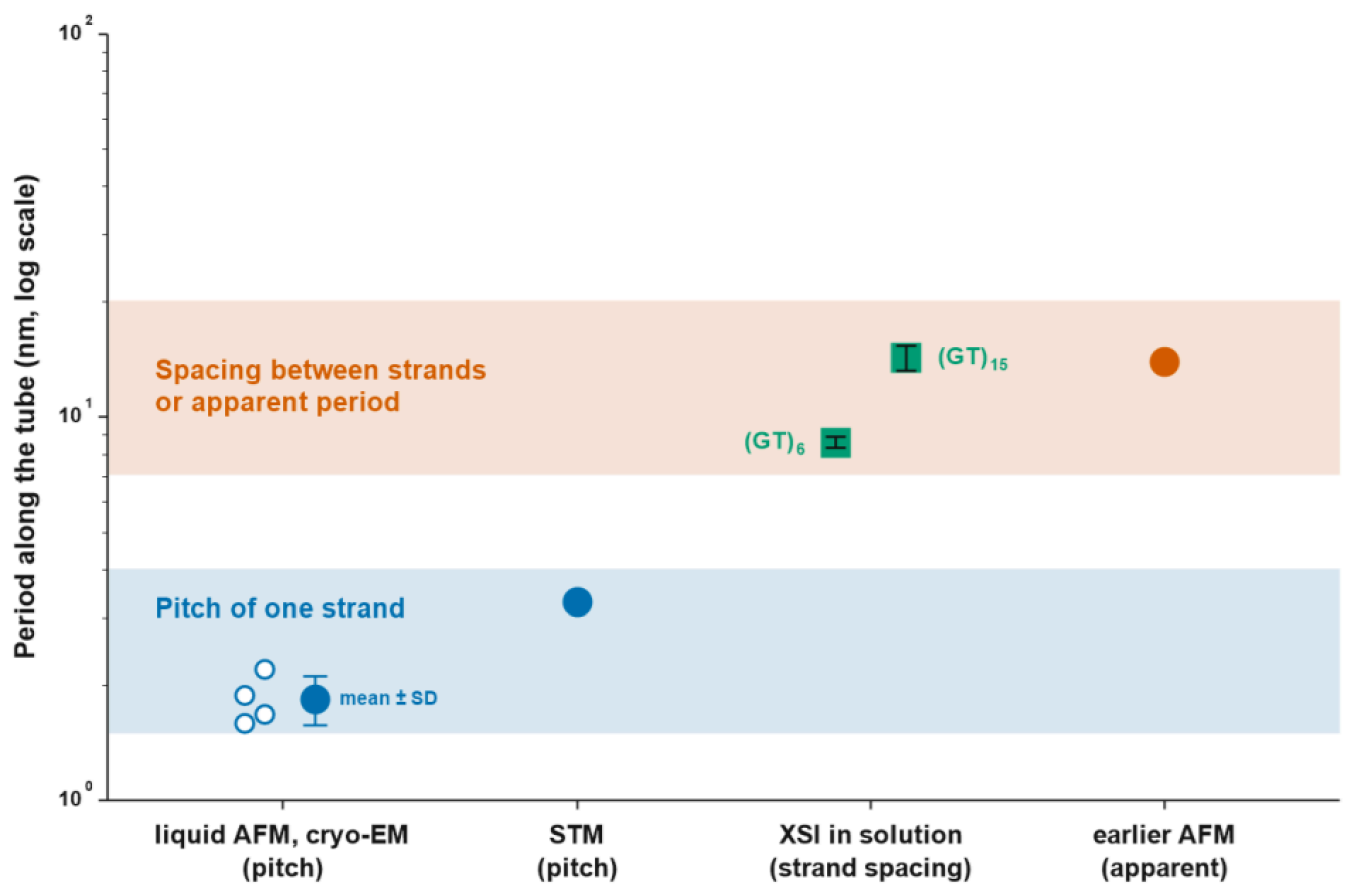
Published periods of ssDNA on SWCNTs measure three different quantities. The first is the pitch of one strand in the dense helix (AFM in liquid and cryo-EM: open circles, the four hybrids for which pitch values are given, 1.59–2.20 nm; filled circle and bar, their mean ± standard deviation, 1.84 ± 0.27 nm, which describes the variation between hybrids [20]; STM, 3.3 nm [35]). The second is the spacing between neighbouring strands measured in solution by X-ray scattering interferometry (XSI; (GT)_6_, 8.6 ± 0.3 nm; (GT)_15_, 14.3 ± 1.1 nm, with error bars as reported [47]). The third is the apparent period of about 14 nm seen in earlier AFM work, which does not depend on the sequence [34]. No uncertainties were reported for the STM and earlier AFM values. All values are taken from the cited published studies and replotted by the authors; no new data are shown.

Any geometric argument also needs the pitch of the DNA helix on the tube. The reported values differ by an order of magnitude (Figure 4), and the difference is systematic and follows the method. High-resolution atomic force microscopy (AFM) in liquid, corroborated by single-particle cryo-electron microscopy (cryo-EM), resolves helices with pitches of 1.59–2.20 nm on five single-chirality hybrids [20]. The pitch depends on the DNA sequence, on the nanotube species and even on the hand of the tube; one sequence wraps (−)(6, 5) with a pitch of 1.68 nm and the mirror-image tube (+)(6, 5) with 1.59 nm [20]. Scanning tunnelling microscopy (STM) of (6, 5) tubes shows strands wrapped at about 63° and repeating every 3.3 nm together with width modulations of 1.9 and 2.5 nm along the strand that the authors did not explain [35].

Earlier AFM work, by contrast, showed a regular pattern with a period of about 14 nm that did not change with the length or the sequence of the DNA [34]. For long genomic single strands, the AFM period is constant along one tube but differs from tube to tube, reaching about 60 nm on one of them [22]. The authors of the high-resolution study attribute such larger values to lower imaging resolution, to imaging of dried samples, or to unsorted tubes wrapped by other sequences [20]. Deposition for AFM is itself sequence-dependent; hybrids made with (GT)_6_ and (GT)_30_ adsorb differently on the substrate, enough to distort the measured length distributions [75]. The nanometre-scale structural pitch and the pattern of ten nanometres or more seen in the earlier AFM work are, thus, different quantities.

Measurements in solution add a third quantity. With gold tags attached to the 5′ ends of the strands, X-ray scattering interferometry shows that (GT)_6_ forms rings stacked along the tube with a spacing of 8.6 ± 0.3 nm. By contrast, (GT)_15_ wraps helically, with a most probable spacing of 14.3 ± 1.1 nm between its strands [47]. These values describe the spacing between neighbouring strands rather than the pitch of one strand, and they respond to conditions. Higher ionic strength compresses the (GT)_15_ spacing from 14.7 to 12.3 nm but hardly changes the (GT)_6_ rings, and dopamine increases both spacings [47]. The solution value for (GT)_15_ is close to the AFM period of about 14 nm, which suggests that some of the longer periods in the earlier work reflect the spacing between strands. The two cannot simply be identified, however, because the spacing in solution depends on the length of the oligomer and the AFM period did not [34,47]. For this reason, the pitch of one strand, the spacing between strands and the apparent period of dried samples (Figure 4) have to be kept apart. Wrapping geometry can be compared with sequence period only within one method, and the experiments proposed below take this into account.

The finite size of the AFM tip contributes to these differences, although it affects widths more than periods. An AFM image is the surface dilated by the shape of the tip, which broadens features whose size is comparable with the tip radius [76]. For a given tip radius, the error grows with the height of the object and is largest for the narrowest features [77], which is the geometry of a strand on a tube about 1 nm wide. The authors of the high-resolution study point out that the radius of an AFM tip is much larger than the expected pitch. The shallow height dips of about 0.2 nm between turns are, therefore, hard to detect and can be masked by a strong tip force. The helix was resolved only with very soft tips in liquid and within a narrow window of imaging force. At lower resolution, pseudo-helices can appear, which, unlike a real helix, change with scan angle and magnification [20]. For a symmetric tip, dilation does not shift the centres of features that the tip resolves, so it cannot, by itself, turn a pitch of about 2 nm into a period of about 14 nm. It can, however, merge turns that the tip cannot separate, and a periodic structure sampled with pixels larger than half its period can appear with a longer, spurious period. Tip convolution thus explains why the structural pitch was not seen in the earlier AFM work [34]. The value of about 14 nm, by contrast, more probably reflects a real modulation on a longer length scale, such as the spacing between strands suggested by the solution data [47] or an effect of drying and deposition [20,75].

The handedness of the wrap was, for a long time, less clear than its pitch. Molecular dynamics has produced both right-handed and left-handed helices for the same kind of strand [66]. Replica-exchange simulations place (TAT)_4_ on its partner (6, 5) tube in a right-handed barrel stabilised by hydrogen bonds between distant bases but (T)_12_ on the same tube in a left-handed wrap [36,37]. All five hybrids examined at high resolution show left-handed helices [20]. This agrees with a simulation study in which the chirality of the DNA, rather than the helicity or handedness of the tube, determines the handedness of the wrap [78]. Simulated strands also adopt several distinct backbone geometries that depend on sequence and tube diameter [68], and short oligonucleotides on a (9, 4) tube divide into ring-like and non-ring conformations [79]. The right-handed barrel predicted for (TAT)_4_ has, therefore, not been confirmed by experiment. The variety of conformations found in simulation also means that one regular helix cannot be assumed for every sequence. Resolving sequences, which purify a single nanotube species from a mixture, form ordered helices, whereas tubes coated with sequences that do not resolve them carry disordered DNA [20].

### 4.4. What Governs the Wrapping

The wrapping depends on the structure of the tube, the primary structure of the DNA, the solution conditions, and time. The complexation of a given recognition sequence depends strongly on the nanotube species and even differs between the two enantiomers of (6, 5), from which the same sequence is displaced by surfactant in different ways [80]. As for the DNA, recognition pairs give better-dispersed, more tightly bound hybrids with a larger zeta potential and greater stability against salt. The amount of DNA on the tube, however, does not correlate with recognition [21], and the stability of (GT)_n_ hybrids is likewise independent of surface coverage [81]. Length matters even at the level of a single nucleotide. Adding or removing one base from a (6, 5) recognition sequence changes the rate of displacement by surfactant about twenty-fold [38]. For short members of the (TAT)_n_ family, the activation energy for removal varies periodically along the series of lengths, repeating every two or three bases [39]. Over a wider range of lengths, the initial rate at which (GT)_3_ displaces surfactant from the tube is more than 300 times that of (GT)_20_. The dependence of the rate on diameter also changes sign for strands longer than six repeats [82]. For short oligonucleotides, binding free energy is the main correlate of kinetic stability, and a tendency to form compact, ring-like conformations adds a smaller positive contribution [79].

Solution conditions also shape the wrap. When the ionic strength is lowered from 500 to 1 mM, more than 25 different oligonucleotides undergo a reversible transition in which the DNA coverage of the surface decreases and the fluorescence quantum yield drops by up to 90% [42]. In solution, the spacing between adsorbed (GT)_15_ strands shortens as the ionic strength rises [47], and adjusting the ionic strength can also gather the DNA into compact regions that leave bare stretches of the tube about 11 nm long [83]. Screening of the backbone by counter-ions can induce in an adsorbed 30-mer a change analogous to the B-to-Z transition, which red-shifts the emission [29]. Time is the last factor. With salmon DNA, an ordered helical coating took weeks to complete, and the photoluminescence rose fifty-fold only when the monolayer was finished [84]. Storage matters as well, because DNA-wrapped tubes tend to aggregate during storage; freeze-drying with suitable cryoprotectants has been introduced to preserve their emission [85]. Hybrids that are compared must, therefore, be prepared and aged in the same way, a requirement that is easily overlooked when results from different laboratories are put side by side. Once the wrap has formed, the SWCNT is in solution with its covalent structure unchanged, and its fluorescence becomes sensitive to changes in DNA conformation, a sensitivity that sensors exploit [29,86].

Two conditions follow from this section for the question of periodicity. Binding strength depends on base composition and on the exact length of a repeat, and sequences can be compared only when the method, the ionic strength and the age of the hybrids are the same.

## 5. Sequence Recognition of Nanotube Structure

### 5.1. Recognition Sequences and Sorting

Sorting experiments give the most direct evidence that DNA recognises nanotube structure. The first sequence-dependent separation used (GT)_n_ with n = 10–45, which assembles into a helix whose electrostatics depend on tube diameter and electronic type, and so, allows fractionation by anion-exchange chromatography [14]. A search of a far larger sequence space then identified more than twenty short sequences, each of which purified one species, and made it possible to purify all twelve major semiconducting species of a synthetic mixture [16]. About 350 sequences were tested, and those that worked share a pattern of alternating purines and pyrimidines. The same sequences hardly differ in how well they disperse the tubes, which places the selection in the separation step rather than in solubilisation [16]. Aqueous two-phase partition later replaced chromatography. With the right pairing of sequence and polymer system, it purified fifteen single-chirality species [40], and a refined version allows continuous multistep sorting of four species from one dispersion [87]. Simple salts can also control the partition. The cation changes partition in an order close to the Hofmeister series, and a fixed ratio of potassium to sodium allowed milligram-scale isolation of (−)(6, 5) with high enantiomeric purity [50]. The sorting methods and their common basis in differences of solvation have been reviewed in detail [24,25].

Two features of these sequences bear directly on periodicity. The recognition sequences of the first library show a periodic purine–pyrimidine pattern, which the authors proposed can hydrogen bond into a two-dimensional sheet and fold around the tube into an ordered barrel [16]. Such a barrel of hydrogen-bonded strands also accounts for the measured charge density of the hybrids [67]. Recognition is, moreover, related to binding strength. A sequence has a higher activation energy for removal on its partner tube than on other species, and the sequence that recognises (6, 5) gives a more stable hybrid than closely related sequences [38,39]. Near ten nucleotides, a length at which the contour of the strand is comparable to the circumference of the tube, separation becomes extremely sensitive to the length and the ends of the sequence. (GT)_6_ purifies (8, 6) while (GT)_5_G enriches a metallic tube, (CCG)_2_CC selects (8, 7) while (CCG)_4_ selects (9, 4), and (TAT)_4_ selects (6, 5) while its compositional isomer (ATT)_4_ selects (7, 5) [16]. The last two sequences also differ in coating exchange, since the diameter dependence of surfactant displacement is steeper for (TAT)_4_ than for (ATT)_4_, which the authors relate to head-to-tail hydrogen bonding between strands [88].

Diameter alone does not explain recognition. (TAT)_4_ and (CCA)_10_ discriminate in opposite directions between (6, 5) and (9, 1), two tubes with identical diameters but different electronic structures [39]. The authors estimated the lateral energy barrier for a base on the carbon lattice at about 0.01 kT, which is too small for the strand to sense the arrangement of the carbon atoms. They attributed the difference to the electronic properties of the two tubes [39]. Displacement kinetics point the same way. For (GT)_5_, the initial rate differs by a factor of about three between (9, 1) and (6, 5), which have the same diameter, and the authors relate this to the chiral angle [82]. Nor do displacement time constants for a wider set of pairs show a clear correlation with diameter [80].

Table 4 lists the recognition sequences of the original library by tube diameter. Almost all of them are short tandem repeats, with repeat units of two to four nucleotides. Trinucleotide repeats occur across the whole range of diameters, whereas four-nucleotide repeats appear only for tubes of 0.83 nm and above, and the four narrowest species are all purified by trinucleotide repeats. One species, (8, 6), accepts repeats of two, three and four nucleotides, so the length of the repeat unit cannot, by itself, determine the partner. Later studies added recognition sequences that are not built from a repeated di-, tri- or tetranucleotide motif, for example, CTC_3_TC for (7, 6), the most stable pair in one comparative study, and G_2_C_8_GC for the zigzag (10, 0) tube [20,80].

### 5.2. Handedness

The handedness of the tube adds a further variable, one that is lost when only the diameter is discussed. A screen of more than 300 short sequences with palindrome symmetry selected over twenty nanotube structures of defined helicity and handedness, covering all three electronic types [17]. In the same work, one sequence folded in two different ways on the two enantiomers of (6, 5) and gave them different fluorescence intensities and chemical reactivities [17]. Replacing natural D-DNA by its mirror image, L-DNA, inverts the handedness of the tube that is purified, as parity requires [48]. Single enantiomers have also become easier to sort [50], and enantiomeric purity can now be quantified without enantiopure standards. The study that introduced this measurement also found that the responses depend on how the nanotubes were synthesised and purified and differ between nanotube sources [89].

Simulations suggest how a short strand senses the hand of the tube. On the two enantiomers of (7, 5), the bases stacked closer to one enantiomer than to the other, but single nucleotides and dinucleotides showed no enantiomer-dependent orientation, whereas ATT trinucleotides adopted different orientations and sugar arrangements [90]. If this holds in experiment, differences in orientation between the two hands first appear at the level of a trinucleotide, which is also the most common repeat unit among the recognition sequences in Table 4. Sensing shows the same stereoselectivity, since enantiopure hybrids respond differently to the two enantiomers of amino acids [45]. A given sequence thus interacts with one of two mirror-image surfaces rather than with “a (6, 5) tube” in general, and any model that relates sequence to tube geometry has to specify which one.

### 5.3. What Machine Learning Has and Has Not Explained

The sequence space is far too large to search exhaustively, so recognition sequences are now often found by machine learning. Models trained on existing sorting data raised the success rate of finding a recognition sequence from about 10% to more than 50% [43]. A later systematic search raised it above 90% and increased the number of known resolving sequences from the order of 10^2^ to 10^3^. Patterns learned from 5-mers and 6-mers also carried over to longer sequences [18]. A deep learning model trained on 216 experimentally screened sequences, 116 of which purified 17 single-chirality species, was correct in 87.5%, 90% and 70% of its predictions for (6, 5), (6, 6) and (7, 4), respectively [19].

On the sensing side, a library of 1408 DNA–SWCNT photoluminescence responses showed that models combining local sequence features with higher-level molecular properties outperform models based on the molecular properties alone [44]. For serotonin sensors, the DNA sequence mattered more than the nanotube chirality [91], and high-throughput selection from a random library identified both base compositions and base patterns that favour binding [49]. Local sequence features or base patterns thus add information to that carried by overall composition and molecular properties [44,49], although these studies do not show what that information is. Prediction is also limited on the side of the analyte. For a dopamine sensor, regression models trained on 63 small molecules reached mean R^2^ values of only 0.2–0.4, whereas classification into strong and weak responses was more reliable, with a mean F1 score of about 0.8 [92]. These models predict responses without explaining their mechanism, and none of them has tested the repeat period as an explicit variable.

So far, machine learning has been used to find sequences, not to predict how they wrap, and we know of no model that predicts the structure of the wrap from the sequence and the (n, m) indices. General predictors of biomolecular structure, such as AlphaFold 3, now handle complexes of proteins, nucleic acids, small molecules and ions [93]. An extended carbon lattice, however, lies outside the systems on which they were trained, and the sub-nanometre structures of DNA on nanotubes are still too few to train or test such a model [20]. Structures of the wrap, therefore, still come from molecular dynamics [36,37,66,68,79], whose results depend on the force field and on the length of sampling, as the opposite handedness obtained for the same kind of strand shows [66]. Machine-learned force fields aim to combine the accuracy of electronic-structure methods with the speed of classical force fields [94]. They could make longer and more reliable simulations possible, but only if they are trained on reference data for bases on curved carbon surfaces, since data for flat surfaces do not transfer to the tube (Section 4.2). The two approaches have already been combined for binding strength. In the high-throughput selection mentioned above, molecular dynamics showed the role of hydrogen bonding in the adsorbed strands, and a classifier trained on the selected sequences distinguished high-affinity sequences and was made available as an online tool [49].

For the analysis of mechanism, a model is useful only if its inputs can be interpreted. One sensing study shows what such models can already do. Classifiers and regression models were trained on the responses of about a hundred sequences, which had been narrowed down by selective evolution from libraries of the order of 10^10^ sequences [95,96]. These models found five serotonin sensors with a larger fluorescence response than those obtained previously [96]. Methods that attribute each prediction of a model to its input features [97] could turn such models into tests of mechanism. For the question of this review, the period can be coded as an explicit feature, for example, as the fraction of positions at which a base recurs p positions further along the strand. A model that includes this feature can then be compared with one that sees only the composition. If the period does not improve the prediction of sorting or sensing outcomes for sequences held out from training, the existing data show no sign of a periodicity effect. If it does, the sequences responsible become candidates for the controlled experiment of Section 10. Because the published training sets were not designed to vary the period at fixed composition, such an analysis can suggest an effect but cannot establish it.

Machine learning can also choose the experiments. The systematic search for sorting sequences described above was already guided by machine learning [18]. In materials research more generally, an autonomous system that plans, runs and analyses its own experiments learned to grow carbon nanotubes at targeted rates [98]. Similarly, Bayesian optimisation reached better reaction conditions in fewer experiments, and more consistently, than expert chemists [99]. Applied to the periodicity question, the same approach would choose which permutations of a fixed composition to test next, so that the geometric and compositional explanations are separated with as few experiments as possible. The value of such a loop depends on the data from which it learns, so the reporting and data-sharing practices discussed in Section 8 are a precondition for the use of artificial intelligence in this field.

### 5.4. Natural and Genomic DNA on Nanotubes

Only a few studies have used natural DNA, and their results are not consistent. Long single strands cut from λ-phage DNA of effectively random sequence wrap nanotubes in tight helices with regular pitch, irrespective of sequence, provided the complementary strand is removed [22]. Salmon genomic DNA enriched the (6, 5) species to more than 86% from a CoMoCAT mixture, whereas single-stranded (GT)_20_ showed little or no chirality preference in the same study. The accompanying simulations attributed the preference to the double-stranded form [23]. With salmon testes DNA, an ordered helical monolayer formed only over weeks [84]. In solution, nanotubes destabilise GC-rich duplex and triplex DNA and induce a B-to-A transition in poly[dGdC] but not in poly[dAdT] [100]. Carboxyl-modified tubes inhibit duplex association of human telomeric DNA and selectively induce the i-motif of its C-rich strand [64]. The SEIRA study mentioned in the Introduction also reported a change in backbone conformation on binding [15].

Natural DNA thus interacts with nanotubes in a sequence-dependent way even in its double-stranded or folded forms. The telomeric repeat, the most obvious periodic test sequence, also introduces its own folded structures into the experiment. We found no study, including in our search of the work published from 2023 to 2026, that has dispersed nanotubes with a defined tandem repeat from natural DNA as a single strand and determined which species it selects. The repeats tested so far have been synthetic oligonucleotides with a repeat motif. Recent work with natural sequences has used hybridisation instead. A coating that combines a domain anchoring it to the nanotube with a capture domain distinguished the single-nucleotide KRAS G12D mutation in circulating tumour DNA from the wild type in serum [101]. Here, the sequence is read by base pairing rather than by the way it packs on the wall.

### 5.5. Base Spacing at Fixed Composition: Evidence from Covalent Chemistry

To our knowledge, the only experiment that has systematically varied the spacing of bases at fixed composition on a single nanotube species comes from covalent chemistry. Lin et al. used a reaction that cross-links guanine to the nanotube wall and screened 15-mer sequences containing two guanines in a cytosine background, separated by different numbers of cytosines, on one enantiomer of (8, 3) [46]. The Raman bands induced by disorder, the Stokes shift and the photoluminescence linewidth all reached a minimum when the two guanines were separated by seven cytosines. The pattern depended on the tube, since the left-handed and right-handed enantiomers of (6, 5), treated with the same set of sequences, gave different spectral patterns [46]. Cryo-EM showed that the reacted hybrid had a helical structure with a pitch of 6.5 Å, which matched the pitch of an armchair helical line of (8, 3) calculated from its indices. Before the reaction, the same hybrid gave no detectable helical order [46]. Similar control has been obtained with halogenated uracil placed in ordered wraps, and changing the spacers between the reactive bases changed where the defects formed along the tube [102]. Simulations of (GT)_10_ suggest that irregular spacing of the guanines along the axis is one source of the spectral broadening after guanine functionalisation [103].

These results show that the spacing of bases in a sequence can be matched to the lattice of a given tube, but only once a covalent reaction has fixed the bases in place. They do not show that the noncovalent wrap, in which the bases can still move, responds to the same spacing, and the lateral barrier of about 0.01 kT estimated for a physisorbed base [39] argues against it. The covalent experiments are, nevertheless, a useful model for the noncovalent test proposed in Section 7 and Section 10, because they already use the design that such a test needs: fixed composition, variable spacing and one enantiomer.

Taken together, recognition depends on sequence pattern, length, chiral angle and handedness in addition to diameter, and an effect of base spacing at fixed composition has, so far, been shown only for covalently fixed hybrids.

## 6. Optical Readout and Applications

Control at the level of sequence matters in practice because the emission of the nanotube depends on its coating. The band-gap emission shifts and changes intensity with the dielectric environment and with the coverage of the surface. As a result, a conformational change in the adsorbed DNA [29], hybridisation with a complementary strand [30,104] or a change in ionic strength [42] can each be detected in the near infrared. When surfactant displaces recognition DNA from single-chirality tubes, the emission rises by a factor of 1.3 to 14.7, depending on the pair [80]. Plasmonic nanoparticles can increase the emission further in a chirality-dependent way [105], and the signal can be read through fluorescence lifetime as well as intensity [8].

Sensors based on this principle have been developed for neurotransmitters, proteins, nucleic acids and viral antigens [4,5,6,28]. Dopamine increases the emission of particular DNA-wrapped and RNA-wrapped tubes by 58–80% in ensemble measurements [41]. (GT)_6_, which is calculated to form ordered rings around the (9, 4) tube and so to impose a periodic potential on the exciton, gives turn-on responses of up to 3500% [106]. For three hybrids of known structure, the dopamine response increased linearly with the pitch of the DNA helix, which the authors relate to the nanotube surface left uncovered by DNA [20]. Fluorescence stability differs by more than three orders of magnitude between polynucleotide coatings [107]. Other targets include serotonin [91], oxytocin [108], microRNA [104,109], cytokines [110] and the SARS-CoV-2 spike protein [111], and protein aggregation has been monitored through an optical fibre [112]. Tracking of individual nanotubes has mapped the extracellular space around synapses [7], and nanotube transistors decorated with ssDNA show a sequence-dependent electrical response to dopamine [113]. A recent mechanistic study, however, showed that ligand binding alone does not change the emission and that the effect depends on the chemical properties of the ligand [86]. Hence, the coating decides both whether an analyte binds and whether the binding is detected.

Sensors built from sorted single-chirality hybrids are more recent. A (7, 5) tube wrapped by C_3_GC_6_GC_4_ in a periodically ordered structure detected Cu^2+^ at concentrations down to 1.20 pM without interference from 13 other ions [114]. A thymine-rich strand on (8, 4) detected Hg^2+^ down to 0.472 nM in river water and allowed imaging in zebrafish [115]. Arrays of structurally defined DNA–nanotube sensors, read by machine learning, classified 253 serum samples from patients with liver, lung or ovarian cancer and from controls with a mean sensitivity of 89% and a specificity of 96% [51]. Proteins also adsorb on such coatings. In a library of 20 proteins, metalloproteins quenched the emission of (GT)_15_-wrapped tubes by up to 94% compared with 20% for proteins without metal [116].

Nanotubes are also used as carriers. Loaded with DNA, they deliver it into intact plant cells and protect it from nuclease degradation, giving protein expression without integration of the transgene [117]. DNA linkers can also direct the assembly of nanotubes into larger structures [118], including junctions between two tubes of different chirality [119]. On the delivery side, duckweed plants take up plasmid-wrapped nanotubes directly from the growth medium and express the encoded transgenes [120]. Wrapping with DNA removes the main obstacle to such uses, the aggregation of bare tubes in water [13]. Reviews of DNA nanomaterials place these hybrids within a wider family of DNA-based constructs [121]. Larger DNA nanostructures can also organise the tubes. DNA templates have arranged nanotubes into parallel arrays, and field-effect transistors made from them had key transport metrics more than ten times better than those of earlier biotemplated devices; placed in patterned cavities, the arrays were aligned over centimetre scales [122]. In addition, DNA origami can now be folded with a reactive polyamine and then decorated with functional molecules [123], which widens the choice of such templates.

These applications also raise the question of safety, which sensing studies often leave aside. The biological effects of carbon nanotubes vary widely with nanotube type, purity, length, aggregation state, functionalisation and route of administration, so general conclusions are difficult, and each material has to be assessed for its intended use [124]. Aspirated into the lungs of mice, SWCNTs caused acute inflammation followed by granulomas around nanotube aggregates and progressive fibrosis [125]. Findings of this kind led to carbon nanotubes being listed, as a whole class, among chemicals recommended for substitution, a step that many researchers in the field regard as scientifically unjustified because nanotubes differ widely in diameter, length, form and surface chemistry [126]. Individually dispersed, coated tubes behave differently. Intravenously injected SWCNTs functionalised with polyethylene glycol were almost completely cleared from the main organs within about two months, with no toxic effects found by necropsy, histology or blood chemistry [127]. An implanted nanotube sensor encapsulated in alginate caused no immune reaction or other adverse response for more than 400 days [128]. A single-chirality nanotube wrapped by ssDNA and injected intravenously accumulated mainly in the liver and showed short- and long-term biocompatibility by histology, body weight, blood count and markers of organ function [124]. Nanotubes can also be degraded by enzymes such as horseradish peroxidase [129] and the myeloperoxidase of human neutrophils, and tubes degraded in this way caused no inflammatory response in the lungs of mice [130].

For DNA-wrapped tubes, two further points matter. The first is that the coating does not stay as prepared. Proteins of blood plasma and cerebrospinal fluid adsorb on ssDNA–SWCNTs and form a corona of defined composition [131]. In macrophages, short strands are displaced from the tube, so that the length of the strand controls the uptake, optical stability and retention of the hybrids [132]. The hybrid characterised in buffer is, therefore, not necessarily the hybrid that acts in a cell. The second is that safety data are specific to the nanotube, the coating, the dose and the route of administration, and they cannot be transferred from one preparation to another. For sequence-defined hybrids, data on long-term fate are still few, and any periodic sequence intended for use in biological systems will need its own assessment of biocompatibility.

The optical studies discussed above also provide the readout for the central question of this review. If the period of a repeat changes how the strand packs, the emission of the tube offers a direct way to detect it.

## 7. Critical Assessment: Does Periodicity Matter?

We now return to the central problem of this review, the discrimination of natural periodic sequences, and consider each factor in turn (the quantitative values are collected in Table 5). The hydrophobic effect and base stacking drive binding but do not explain which sequence is bound. The affinity of the individual bases reflects composition, and Section 4.2 shows that even its order is uncertain. The question here is whether periodicity can supply what neither of them can, namely, whether a motif that matches the preferred wrapping geometry of a given tube makes the extended coating more ordered. This idea builds on earlier structural models of recognition. Tu et al. proposed that the periodic purine–pyrimidine pattern of recognition sequences lets them form a hydrogen-bonded two-dimensional sheet, which folds selectively on the matching tube into an ordered three-dimensional barrel [16]. Replica-exchange simulations by Roxbury et al. later found ordered wraps stabilised by hydrogen bonds between bases with a structure that depends on the DNA sequence [36] and on the tube [37]. Applied to natural repeats, these models raise the possibility that the period of the motif, rather than the individual base, sets chiral selectivity, so that a SWCNT could separate repeats that look similar at first glance. The published evidence for and against this possibility is assessed below.

Several observations are consistent with this interpretation. Recognition sequences show a periodic purine–pyrimidine pattern [16]. Binding strength changes sharply when one nucleotide is added or removed, and it varies periodically along a length series within one family of repeats [38,39]. Compositional isomers with the same repeat length select different tubes [16]. In simulation and at high resolution, recognition sequences form ordered, hydrogen-bonded wraps whose pitch depends on both sequence and tube, while non-resolving sequences give disordered coatings [20,36,37]. The authors of the structural study also remark that a coating which is not commensurate with the nanotube lattice may vary along the tube, and they suggest this as the reason atomic resolution was not reached [20]. Ordered (GT)_6_ rings are calculated to impose a periodic surface potential that changes the exciton lifetime [106], and rings of (GT)_6_ stacked at regular intervals along the tube have now been observed in solution [47]. In covalent chemistry, a fixed spacing of two guanines gives an ordered defect array on one (8, 3) enantiomer, with a period that matches the lattice of that tube [46] (Section 5.5). Finally, machine learning studies of sorting and sensing find that short sequence patterns carry predictive information [18,44], and base patterns as well as composition govern binding affinity [49].

Other observations, however, contradict it. The long-period pattern seen in earlier AFM work is the same whatever the sequence [34], and genomic strands of random sequence wrap as regularly as designed repeats [22], so a periodic coating does not need a periodic sequence. The canonical repeat (GT)_20_ showed almost no chirality preference in a direct comparison with genomic DNA [23]. Sequences discriminate between tubes of the same diameter [39] and between the two hands of one tube [17,80], which a rule based on diameter and repeat period alone cannot reproduce. A sequence that is not built from a repeated motif forms one of the most stable pairs reported [80]. The lateral energy barrier between a base and the carbon lattice has been estimated at about 0.01 kT per base, which argues against registry with the atomic lattice as the origin of recognition [39]. In solution, the spacing between (GT)_n_ strands changes with the length of the oligomer at the same motif [47], so the period of the coating is not set by the period of the sequence alone. In the covalent hybrids, too, the order appears only after the reaction, and the same sequence on the same tube gave no detectable helical order before it [46]. For at least one analyte, moreover, the sensor response depends more on the sequence than on the tube [91].

For noncovalent wrapping, then, the evidence on both sides is indirect. To our knowledge, no published study has varied the period at fixed composition, chirality, and handedness, and the one study with this design concerns covalently fixed hybrids (Section 5.5). A purely compositional explanation is, therefore, still possible, and only a controlled experiment with controls matched for length and composition can separate it from the geometric one. The published affinities and pitches also come from different methods under different conditions and should be compared with caution (Section 4.2 and Section 4.3).

This caution applies to the whole of Table 5, which brings together values from different studies that are comparable only within narrow limits. The rows are, therefore, grouped by the kind of quantity. Values in different groups describe different properties and cannot be combined. Within a group, values obtained by different methods often measure different things even when they share a unit. The pitches of 1.59–2.20 nm, the STM period of 3.3 nm, the spacing between strands in solution and the AFM pattern of about 14 nm are distinct quantities (Section 4.3). Likewise, the free energies per nucleotide of homopolymers measured by force spectroscopy in water [74] differ from the binding energies of single bases calculated by DFT in vacuum [70] in the state of the base, in the medium and in the reference state. Even a single method gives values that depend on the sample. The source, purification and enantiomeric purity of the nanotubes [89]; the length of the DNA [47,82]; the ionic strength [42,47]; the age of the hybrids [84] and the deposition of samples for imaging [75] all change the results. These conditions differ between the studies in the table. The safest comparisons are those made within one study under one set of conditions, such as (GT)_6_ against (GT)_15_ in solution [47], (GT)_3_ against (GT)_20_ in displacement kinetics [82] or the four homopolymers on one surface [74]. Ratios between values in different rows or from different studies are, at best, order-of-magnitude estimates, and a common reporting standard (Section 8.2) would be needed before such data could be pooled.

The optical studies suggest a consequence that can be tested. NIR fluorescence persists and is modulated as DNA changes conformation [29,41,86], so an ordered coating should give a narrower, more reproducible spectral signature than a disordered one. The sharpening of the absorption peaks on completion of an ordered DNA monolayer points the same way [84]. The covalent experiments agree, since the guanine spacing that gave the least disorder also gave the narrowest photoluminescence line [46], and simulations suggest that irregular spacing of the guanines along the axis broadens the spectra after guanine functionalisation [103].

The geometric interpretation has to be weighed against its main alternative. In the compositional view, selectivity is set only by the composition of the motif (the G/A/T/C ratio and chain flexibility), and geometric register plays no role. Both views predict a sequence-dependent response, but they differ on one testable point. If composition decides, the response should follow composition and, at fixed composition, be indifferent to the period of the motif; if geometry decides, the tubes should separate even motifs of identical composition but different period. The decisive experiment therefore compares motifs matched for composition and length but differing in period (permutation controls) across tubes of known (n, m) and handedness. Responses that diverge between tubes at fixed composition favour a geometric origin, and responses that do not favour a compositional one. The covalent study of Section 5.5 shows that such a design is practical, since a series of 15-mers of fixed composition and variable spacing was screened on one enantiomer [46]. The experiment has two limitations. Changing the period at fixed composition and length still alters the local order of base packing, so period and composition never fully separate, and a permuted motif of identical composition is needed as an additional control. In addition, the telomeric motif TTAGGG (period 6) is G-rich and prone to G-quadruplex folding, so any comparison at period 6 should also include a motif that is not G-rich.

Four confounding factors have to be controlled. One is folding of the sequence itself. G-rich repeats, telomeric TTAGGG among them, can fold into quadruplexes [63], and nanotubes are known to induce the i-motif in telomeric DNA [64]. Either structure reshapes both the packing and the optical response, and so, may mask or mimic an effect of period, which is why such sequences need companion controls in which folding is disrupted. Another is the long-period pattern of the earlier AFM work. As Section 4.3 and Figure 4 show, the value of about 14 nm [34] does not depend on the sequence and should not be compared with sequence period. Only the structural pitches from high-resolution AFM, cryo-EM and STM [20,35] are relevant. Preparation history matters as well, since ionic strength and ageing change coverage and emission by an order of magnitude or more [42,84]. The last is the purity of the enantiomer, which can vary with the source of the nanotubes and can now be measured directly [89]. If any of these factors are neglected, a geometric origin may be confirmed or rejected for the wrong reason.

## 8. Reproducibility, Standardisation and Open Data

The questions raised in Section 7 can be answered only if results from different laboratories can be compared. We examine, in turn, whether such comparisons are possible at present (Section 8.1), which standards would help (Section 8.2) and how the data of the field could be shared (Section 8.3).

### 8.1. Reproducibility Between Laboratories

Would two laboratories that use the same sequence and the same nanotube species obtain the same wrap and the same recognition? We found no published interlaboratory comparison of DNA–SWCNT hybrids, so the answer rests on indirect evidence and depends on the level considered. The preference of a sequence for a nanotube species seems to be reproducible. Sequence-dependent sorting has been achieved with two different separation principles, ion-exchange chromatography [14,16] and aqueous two-phase partition [40,87], which suggests that the preference belongs to the hybrid rather than to one separation method. Quantitative signals are much less certain. Chirality-pure hybrids can be up to ten times brighter than their unsorted counterparts, residual surfactant from the preparation impairs their response, and the DNA sequence sets their colloidal stability and selectivity [133]. The responses of sorted nanotubes also differ between nanotube sources [89]. Sonication shortens the tubes in a time-dependent way [134], ionic strength and ageing change coverage and emission by an order of magnitude or more [42,84], and the deposition of hybrids for AFM depends on the sequence [75]. For the structure of the wrap, the question cannot yet be answered, because the sub-nanometre structures come from a single study [20], and the earlier structural periods differ by an order of magnitude between methods (Section 4.3). The same sequence applied to the same nanotube material can, therefore, be expected to select the same species in another laboratory but not to give the same intensities, kinetics or pitches unless the material and its preparation are matched.

### 8.2. Standardised Characterisation and the Efficiency of Wrapping

Agreed methods would make such comparisons possible. For the nanotubes themselves, standards and reference procedures exist. Harmonised sample-preparation protocols were proposed to make the characterisation of SWCNTs repeatable and reproducible between laboratories [135]. In addition, a technical specification describes how semiconducting species and their relative abundance are determined by near-infrared photoluminescence [136]. Directly measured absorption cross-sections allow the concentrations of individual species to be calculated from absorption spectra [137], and the enantiomeric purity of sorted material can now be measured [89]. For the hybrid, the situation is less settled. The term wrapping efficiency, or encapsulation efficiency, is used for at least three different quantities: the fraction of the nanotubes brought into stable dispersion, the amount of DNA bound per tube or per unit length, and the fraction of the tube surface that the DNA covers. These are measured in different ways and need not agree with each other or with recognition. The amount of DNA on the tube does not correlate with recognition [21], and the stability of (GT)_n_ hybrids is independent of surface coverage [81]. The route of preparation matters as well. Direct sonication shortens the tubes [134] and cannot preserve secondary structures of the DNA [138], which matters for repeats that fold (Section 3.2). Exchanging a bile-salt coating for DNA, by contrast, takes about 10 min, converts more than 90% of the tubes into the DNA-wrapped form and can be applied to presorted species [138], although residual surfactant must then be removed [133]. A general minimum-information standard has been proposed for research at the bio–nano interface, covering material characterisation, biological characterisation and experimental protocols [139]. Table 6 adapts it to ssDNA–SWCNT hybrids as a minimum set of items that would make results from different laboratories comparable and allow them to be pooled.

### 8.3. Open Data and a Shared Database

The field also lacks a common place for its data. We are not aware of any public database of DNA–SWCNT hybrids. Sorting and sensing results are reported in the supporting information of individual papers, often only for the successful sequences and in formats that differ between laboratories. Even a large screen may provide its data only on request while offering its trained model as an online tool [49]. A shared database nevertheless appears feasible. Screens of 10^2^–10^3^ sequences with experimental readouts already exist [18,19,44]; infrastructure for nanomaterial data has been built with ontologies, open standards and programmatic access [141]; and a structural database has recently been created for short tandem repeats themselves [142]. A database for DNA–nanotube hybrids would hold, for each sequence and nanotube species, the items of Table 6 together with the outcome: whether the species was resolved, binding and displacement data, the structural pitch with the method used, and the optical response with the raw spectra. Three features would make it useful for the question of this review. Negative results, sequences that failed to sort or to respond, should be included, because a model trained only on successes cannot learn what separates them from failures. Each value should carry its method and conditions, so that quantities from different methods are not mixed (the problem discussed above for Table 5). The data and the code used to analyse them should also follow the FAIR principles, that is, be findable, accessible, interoperable and reusable [140]. A realistic first step would be for authors to deposit the complete screening data behind each sorting or sensing study, including negative results, in a public repository in a common format, from which a database could later be assembled.

## 9. Conclusions

Several points can be considered established. ssDNA forms ordered wraps on a SWCNT, mostly helices with a structural pitch of a few nanometres and, for some short strands, stacked rings. The roles of the forces behind the wrapping are known in outline: base stacking and the hydrophobic effect provide the driving force, the charged backbone sets the spacing and gives colloidal stability, and hydrogen bonding orders the wraps of recognition sequences. Short sequences can also recognise single nanotube species, including their handedness. In contrast, two points that are often treated as established are not. One is the order of base affinities on a nanotube; the other is the meaning of the long-period pattern seen in earlier AFM work, which differs from both the pitch of one strand and the spacing between strands in solution. The handedness of the wrap was uncertain for a long time, but it now appears to be left-handed for natural D-DNA [20,78]. Reproducibility between laboratories has not been tested directly, and the field still lacks a common reporting standard and a shared database (Section 8). Table 7 summarises the state of the evidence. For noncovalent hybrids, the central question is still open and requires a direct test: whether a SWCNT of given geometry can distinguish natural periodic sequences by the way they pack.

## 10. Future Directions

The most direct next step is a controlled comparison of natural periodic sequences (the two complementary strands of the (CA)_n_ microsatellite, (CA)_n_ and (GT)_n_; the telomeric repeat TTAGGG; and minisatellite motifs) with synthetic controls matched for length and composition. It should use tubes of known (n, m) and handedness, with the enantiomeric purity of each sample measured [89], and can follow the design already used for covalently cross-linked hybrids [46]. Binding strength would be measured by surfactant displacement [38,39], wrapping pitch by a single structural method, optical response by absorption and photoluminescence, and packing energetics by molecular dynamics. If the tubes distinguish such sequences, this would open a route to sequence-encoded nanosensing of genomic repeats and to studies of chromatin organisation. If they do not, the selectivity most probably has a compositional origin. The proposed steps are summarised in Table 8.

Further steps would make this test more decisive. G-rich and C-rich repeats need companion controls with their folded structures disrupted, so that folding is not mistaken for an effect of period. Wrapping pitches should be reported from structural methods under stated preparation conditions (Table 6), because with the present order-of-magnitude spread, values from different techniques cannot be compared. Finally, the geometric fit between a repeat period and the circumference of the tube should be put in quantitative form and anchored to the sub-nanometre structures [20], so that the comparison produces numerical predictions. This treatment is outside the scope of the present review.

The question also bears on genome structure. The copy number of a tandem repeat varies between individuals, and expansions of short tandem repeats cause a range of neurological disorders [61], while telomeric repeats fold into quadruplex and i-motif structures [63,64]. Three-dimensional structures of short tandem repeats implicated in neurodegenerative diseases and cancers, alone and in complexes with proteins or ligands, have recently been collected in a dedicated database [142]. At present, such features are read by sequencing or hybridisation, as in the recent nanotube sensor for a point mutation [101]. A nanotube whose emission depended on the period and length of a repeat would add a label-free optical readout of repeat structure, but whether this is possible depends on the experiment proposed above.

## Figures and Tables

**Table 1:** Main stages in the study of hybrids of single-stranded DNA (ssDNA) and single-walled carbon nanotubes (SWCNTs).

Stage	Period	Main Advances	Sources
1	2003	ssDNA found to disperse SWCNTs in water; helical wrapping by π-stacking proposed; (GT)_n_ hybrids separated by diameter and electronic type; backbone changes on binding seen by surface-enhanced infrared absorption (SEIRA)	[13,14,15]
2	2006–2009	Genomic DNA shown to wrap nanotubes and to enrich one species; DNA conformation and hybridisation read through the near-infrared (NIR) emission; base affinities calculated and measured; pitch measured by atomic force microscopy (AFM) and scanning tunnelling microscopy (STM); recognition sequences found for twelve semiconducting species	[16,22,23,29,30,31,32,33,34,35]
3	2011–2017	Ordered, hydrogen-bonded wraps described by simulation; recognition tied to binding strength; sorting by aqueous two-phase partition; control of handedness; neurotransmitter sensors; ionic-strength transition of the adsorbed DNA	[17,36,37,38,39,40,41,42]
4	2018–2022	Machine-learning search for sorting and sensing sequences; stereoselective sensing; DNA-guided covalent modification in register with the nanotube lattice	[18,43,44,45,46]
5	2023–2026	Sub-nanometre structures of the wrap by AFM in liquid and cryo-electron microscopy (cryo-EM); spacing of strands measured in solution; mirror-image sorting with L-DNA; high-throughput selection of high-affinity sequences; deep learning prediction of sorting sequences; salt-driven sorting of enantiomers; sensor arrays read by machine learning	[19,20,47,48,49,50,51]

**Table 2:** The three contributions to ssDNA–SWCNT binding compared.

Criterion	π–π Stacking (Adsorption)	Helical Wrapping (Conformation)	Electrostatic Stabilisation
What it is	Aromatic rings of the bases lie flat on the wall	The sugar–phosphate backbone bends and winds around the tube at an angle	Charged backbones of neighbouring tubes push each other apart
Driving force	Hydrophobic effect with π-orbital overlap	Electrostatic and torsional interactions within the backbone; hydrogen bonding between bases	Coulomb forces and the solvent’s ionic cloud
Sensitivity to base	Depends on the base; the order differs between methods (Table 3)	Set by the sequence pattern and by hydrogen bonding between bases	Fixed by total PO_4_^−^ charge; the same whatever the base
Role of chirality	Weak; the bases adsorb on every species	Central: ordered wraps are specific to tube species and handedness	Indirect, through the charge density of the hybrid
Effect on the tube	Changes the dielectric environment and, with it, the emission	Holds individual tubes apart while keeping fluorescence	Confers colloidal stability
Sources	[13,68]	[20,36,37,66]	[42,67,69]

**Table 4:** Recognition sequences that purify the twelve major semiconducting species ordered by tube diameter (data compiled from [16]). Diameters are calculated from the indices (n, m).

Species (n, m)	Diameter, nm	Recognition Sequences	Repeat Unit, Nucleotides
(9, 1)	0.75	(TCC)_10_, (TGA)_10_, (CCA)_10_	3
(6, 5)	0.75	(TAT)_4_, (CGT)_3_C	3
(8, 3)	0.77	(TTA)_4_TT, (TTA)_3_TTGTT, (TTA)_5_TT	3
(7, 5)	0.82	(ATT)_4_, (ATT)_4_AT	3
(8, 4)	0.83	(ATTT)_3_	4
(10, 2)	0.87	(TATT)_2_TAT	4
(7, 6)	0.88	(GTT)_3_G, (TGT)_4_T	3
(9, 4)	0.90	(GTC)_2_GT, (CCG)_4_	3
(8, 6)	0.95	(GT)_6_, (TATT)_3_T, (TCG)_10_, (GTC)_3_, (TCG)_2_TC, (TCG)_4_TC, (GTC)_2_	2, 3 and 4
(9, 5)	0.96	(TGTT)_2_TGT	4
(8, 7)	1.02	(CCG)_2_CC	3
(10, 5)	1.04	(TTTA)_3_T	4

**Table 5:** Quantitative values for the ssDNA–SWCNT system quoted in this review as reported in the cited sources and grouped by the kind of quantity. Values are comparable only within a group and, within a group, only when they were obtained by the same method under the same conditions (Section 7). Group headings are shown in italics.

Parameter	Value/Range	System and Method	What It Tells Us	Source
*Lengths along the tube*				
Structural pitch of the DNA helix	1.59–2.20 nm	AFM in liquid and cryo-EM, five single-chirality hybrids	Depends on sequence and tube species; left-handed helices	[20]
Coiling period; wrapping angle	3.3 nm; ≈63°	STM, (6, 5) tubes	Structural pitch on one species	[35]
Surface pattern	≈14 nm	AFM, earlier work	Independent of DNA length and sequence	[34]
Spacing between adjacent strands in solution	8.6 ± 0.3 nm, (GT)_6_ rings; 14.3 ± 1.1 nm, (GT)_15_ helices	X-ray scattering interferometry, gold-tagged strands	Spacing between strands, not pitch; depends on length and ionic strength	[47]
Helical period after DNA-guided covalent cross-linking	0.65 nm (6.5 Å)	Cryo-EM, C_3_GC_7_GC_3_ on one (8, 3) enantiomer	Matches an armchair helical line of the lattice	[46]
*Charge and binding energies*				
Linear charge density of poly(GT) hybrids	6.0 e/nm on (6, 5); 6.8 e/nm on (7, 5)	Capillary electrophoresis	Mobility comparable to double-stranded DNA; constrains structural models	[67]
Binding free energy per nucleotide, SWCNT	38.1 (A), 33.9 (G), 23.3 (T), 17.1 (C) kT	Single-molecule force spectroscopy	The curved wall binds far more strongly than graphite	[74]
Binding free energy per nucleotide, graphite	11.3 (T), 9.9 (A), 8.3 (G), 7.5 (C) kT	Single-molecule force spectroscopy	Order differs from that on the nanotube	[74]
Base binding energy on graphene and boron nitride	0.54–0.75 eV (LDA); 0.93–1.18 eV (PBE + vdW)	DFT, vacuum	Absolute value depends on the functional	[70]
*Sorting and recognition*				
Recognition sequences	>20 out of about 350 tested, for 12 semiconducting species	Ion-exchange chromatography	Each sequence purifies one species	[16]
Single-chirality species purified	15	Aqueous two-phase partition	Partition is sequence-dependent	[40]
Structures of defined handedness	>20 from >300 sequences screened	Aqueous two-phase partition	DNA recognises handedness	[17]
Success rate of the search for recognition sequences	≈10% raised to >50% and then to >90%	Machine learning with experimental test	Sequence patterns can be learned	[18,43]
Resolving sequences in one screen	116 of 216 for 17 species; prediction accuracy 70–90%	Aqueous two-phase partition and deep learning	Large training sets now exist	[19]
Enrichment of (6, 5) by salmon genomic DNA	>86%	Photoluminescence and NIR absorption	Natural DNA can select one species	[23]
*Optical and kinetic responses*				
Emission increase on displacing DNA by surfactant	1.3- to 14.7-fold; time constants 9–230 s	Single-chirality recognition pairs	Stability and brightness depend on the pair	[80]
Initial rate of surfactant displacement by (GT)_n_	(GT)_3_ more than 300 times that of (GT)_20_; about 3-fold difference between (9, 1) and (6, 5)	Time-resolved NIR fluorescence	Rate depends on length and chiral angle as well as on diameter	[82]
Loss of quantum yield at low ionic strength	up to 90% between 500 mM and 1 mM	Photoluminescence, more than 25 oligonucleotides	DNA coverage changes with ionic strength	[42]
Emission increase on completing a DNA monolayer	≈50-fold, 20–50 days after preparation	Photoluminescence and high-resolution electron microscopy, salmon testes DNA	Ordering of natural DNA on the wall is slow	[84]

**Table 6:** Proposed minimum reporting set for ssDNA–SWCNT hybrids adapted from a general standard for bio–nano research [139].

Item	What to Report	Why It Matters
Nanotube material	Source and batch, synthesis method, purification and sorting steps	Responses of sorted nanotubes differ between sources [89].
Species composition	Species and their relative abundance by photoluminescence or absorption; species concentrations from absorption cross-sections; enantiomeric purity of sorted material	Recognition can be assigned to one species and one hand [89,136,137].
DNA	Full sequence, length, purity and supplier; for natural DNA, the source and how the single strand was obtained	Binding depends on length down to one nucleotide; long DNA disperses tubes only as a single strand [22,38,39].
Preparation	Sonication energy and time or surfactant-exchange conditions; DNA-to-nanotube ratio; centrifugation; removal of free DNA and residual surfactant	Sonication shortens tubes; residual surfactant impairs sensing [133,134,138].
Bound DNA	Which quantity is reported (dispersion yield, DNA per unit length or surface coverage) and how it was measured	The quantities called wrapping efficiency are not interchangeable [21,81].
Measurement conditions	Buffer, ionic strength, pH, temperature and age of the sample	Coverage, spacing and emission change with ionic strength and time [42,47,84].
Tube length	Length distribution and the method used; deposition conditions for AFM	Deposition for AFM depends on the sequence [75].
Data and code	Raw spectra, images and analysis code in a public repository, including negative results	Allows reanalysis and pooling (Section 8.3) [140].

**Table 7:** Summary of the evidence: what is established, what is disputed and what is still open.

Question	Status	Main Evidence	Sources
Does ssDNA form ordered wraps on SWCNTs?	Established	Helices resolved by AFM in liquid, cryo-EM and STM; rings of (GT)_6_ and helices of (GT)_15_ in solution	[20,35,47]
Which forces hold the wrap?	Established in outline	Stacking and the hydrophobic effect bind; backbone charge sets the spacing; hydrogen bonds order recognition wraps	[13,36,37,67,69]
Handedness of the wrap	Largely settled for natural DNA	Left-handed in all five resolved structures; in simulation, the chirality of DNA sets the hand	[20,78]
Do short sequences recognise species and enantiomers?	Established	Single-chirality and single-enantiomer sorting; L-DNA inverts the hand that is selected	[16,17,48,50]
Order of base affinities on a nanotube	Disputed	Order depends on the method and on a flat or curved surface (Table 3)	[32,74]
Meaning of periods of 10 nm or more	Disputed	Not the pitch of one strand; in solution, partly the spacing between strands; the AFM period did not depend on length; conventional AFM tips cannot resolve the pitch	[20,34,47]
Is the diameter the recognised property?	Not supported	Tubes of equal diameter are distinguished; chiral angle and handedness matter	[17,39,82]
Do machine learning models explain recognition?	Predictive, not mechanistic	High success in finding sequences; sequence patterns add information to composition; the mechanism is not identified; no model predicts the structure of the wrap	[18,19,43,44,92]
Does base spacing at fixed composition matter?	Shown only for covalent hybrids	Least disorder at one guanine spacing on (8, 3) after cross-linking; not tested for noncovalent wraps	[46]
Are results reproducible between laboratories?	Not tested directly	No interlaboratory comparison found; species preference holds across separation methods; intensities, kinetics and responses depend on nanotube source, purity and preparation; sub-nanometre structures from one study	[20,40,89,133]
Which species does a tandem repeat from natural DNA select as a single strand?	Open	No study found up to September 2026 (Section 5.4)	–

**Table 8:** Proposed steps for testing whether sequence periodicity affects noncovalent ssDNA–SWCNT recognition.

Step	Design and Controls	Readout	What the Outcome Would Show
1. Compare natural repeats with matched controls.	(CA)_n_ and (GT)_n_ strands, the telomeric repeat TTAGGG and minisatellite motifs, each against synthetic controls matched for length and composition; tubes of known (n, m) and handedness, with the enantiomeric purity of each sample measured [89]	Surfactant displacement [38,39]; absorption and photoluminescence	A response that differs between tubes at fixed composition favours a geometric origin; no difference favours a compositional one.
2. Separate period from composition.	Permuted motifs of identical composition and length (permutation controls), following the design used for covalently cross-linked hybrids [46]	As in step 1	If the response follows the period rather than the composition, the period itself is read by the tube.
3. Exclude folding.	Companion controls for G-rich and C-rich repeats with quadruplex or i-motif folding disrupted [63,64]; at period 6, a motif that is not G-rich	As in step 1, compared with the folding-disrupted controls	Folding is not mistaken for an effect of period.
4. Measure the wrap consistently.	Structural pitch from one high-resolution method with preparation, ionic strength and ageing stated [20,42,84]	Pitch for each sequence and tube	Pitches become comparable between sequences.
5. Make the predictions quantitative.	Geometric fit between repeat period and tube circumference anchored to the sub-nanometre structures [20]; packing energetics by molecular dynamics	Predicted ranking of tubes for each repeat	The comparison becomes a test of numerical predictions.

## Data Availability

No new data were created in this study. All values shown in Figure 3 and Figure 4 and in Table 3, Table 4 and Table 5 are compiled from the published literature, with their sources given in the captions and tables. The statistical comparisons in Section 4.2 were calculated by the authors from these published values.

## Abbreviations

The following abbreviations are used in this manuscript:

AFM	atomic force microscopy
cryo-EM	cryo-electron microscopy
DFT	density-functional theory
DFT-D2, DFT-D3	Grimme’s empirical dispersion correction, versions 2 and 3
FAIR	findable, accessible, interoperable and reusable
LDA	local-density approximation
MP2	second-order Møller–Plesset perturbation theory
NIR	near-infrared
PBE	Perdew–Burke–Ernzerhof functional
qM	quasi-metallic
SC	semiconducting
SEIRA	surface-enhanced infrared absorption
ssDNA	single-stranded DNA
STM	scanning tunnelling microscopy
SWCNT	single-walled carbon nanotube
TS	Tkatchenko–Scheffler dispersion scheme
vdW	van der Waals
vdW-DF, vdW-DF2	van der Waals density functional, versions 1 and 2
XSI	X-ray scattering interferometry
